# Practical Notes and Formulæ

**Published:** 1882-12-20

**Authors:** 


### PRACTICAL NOTES AND FORMULÆ.

**Diarrhœa Pills.**—Prof. William Thomson, of the University of the City of New York, recommends the following as a remedy for diarrhœa—

℞ Plumbi acetatus........................... grs. xvi,
Pulv. camphoræ........................... grs. xij,
Pulv. opii................................ grs. iij,
Bismuth subcarb.......................... grs. xij,
Ext. gentian.............................. q. s.

Make into twelve pills.

Dose, one pill every hour to three hours, according to severity of disease.—*New Remedies.*

**Soap Liniment.**

℞ Oleic acid...................................... ℥ ij,

Bicarbonate sodium.............................. ʒ v,

Camphor......................................... ℥ ij,

Oil rosemary.................................... ℥ ss,

Water........................................... ℥ vi,

Alcohol......................................... O ij.

Dissolve the camphor in the alcohol, add the oleic acid and the oil of rosemary, then the soda gradually, and when effervescence has ceased, add the water, and filter. This will not deposit in cold weather.—*Geo. R. Martin, Proc. Penn. Phar. Association.*

**Algerian Corn Cure.**—Dr. Barbier speaks highly of the subjoined—

Acetic acid.................................. 1 ounce,
Iodine....................................... 37 grains,
Alcohol...................................... 1 ounce.

Dissolve the iodine in the alcohol and add the acetic acid. A few drops of the liquid are to be rubbed on the corn morning and evening, so as to gradually dissolve it.

We published some time ago a corn remedy which had been recommended by a Russian, M. Gezow. Dr. Traill Green, writing in the Medical and Surgical Reporter, expresses the utmost satisfaction with this formula, which he has used in numerous cases of both hard and soft corns with unvarying success. The formula is as follows—

Salicylic acid...................... 30 parts, or grs. xxx,
Ext. cannabias indica................ 5 parts, or grs. v,
Collodion.......................... 240 parts, or f. ℥ ss.

—*Boston Jour. of Chem*

**A Stimulating Expectorant.**—Dr. Fothergill commends the following—

Am. carbonat . . . . . . . . . . gr. v,
Tinct. nux. vom . . . . . . . . . . m. x,
Tinct. scillæ . . . . . . . . . . ℥ ss,
Inf. serpentar . . . . . . . . . . ℥ i.

Sig. Three times daily.—*Boston Jour. of Chem.*

**Tape Worm.**—Salicylic acid in full doses, followed by ol. ricin. com., is said to expel tape worm when all other remedies fail.—*Louisville Med. News.*

**Pills for Constipation.**—

℞ Quiniæ sulphatis . . . . . . . . . . grs. xv,

Piperinæ . . . . . . . . . . } aa grs. xij,

Hydrarg. submuriat . . . . . . . . . . }

Ext. nucis vomic . . . . . . . . . . grs. iv.

M. ft. in pil. No. xxx.

M. S. One pill morning and evening.—*N. O. Med. and Surg. Journal.*

THE AROMATIC POWDER OF CHALK AND OPIUM—Is prepared as follows:

℞ Aromatic powder of chalk . . . . . . . . . . 9¾ ounces,

Opium . . . . . . . . . . ¼ ounce,

Formula for aromatic powder of chalk:

℞ Cinnamon . . . . . . . . . . 4 ounces,
Nutmeg . . . . . . . . . . 3 ounces,
Saffron . . . . . . . . . . 3 ounces,
Cloves . . . . . . . . . . 1½ ounces,
Cardamon seed . . . . . . . . . . 1 ounce,
Refined sugar . . . . . . . . . . 25 ounces,
Prepared chalk . . . . . . . . . . 11 ounces.

M. Triturate thoroughly and pass through a fine seive. Dose, 10 to 60 grains.

**Naquet's Hair-Dye, Modified.**—

Citrate of bismuth . . . . . . . . . . 1 ounce,
Ammonia, sufficient.
Hyposulphite of soda . . . . . . . . . . ½ ounce,
Alcohol . . . . . . . . . . 3 ounces,
Or, glycerine . . . . . . . . . . ½ ounce,
Water to complete . . . . . . . . . . 16 ounces.

Rub the citrate into a thin paste with lukewarm water, and add enough ammonia to make a solution in which the hyposulphite is to be dissolved. Filter, and complete the measure, adding the alcohol last, if it is employed as a preservative.—*Drug. Cir.*

